# The VEGF-A exon 8 splicing-sensitive fluorescent reporter mouse is a novel tool to assess the effects of splicing regulatory compounds *in vivo*

**DOI:** 10.1080/15476286.2019.1652522

**Published:** 2019-08-21

**Authors:** M. Stevens, E. Star, M. Lee, E. Innes, L. Li, E. Bowler, S. Harper, D. O. Bates, S. Oltean

**Affiliations:** a Institute of Biomedical and Clinical Science, Medical School, College of Medicine and Health, University of Exeter, Exeter, UK; b Bristol Renal, School of Clinical Sciences, University of Bristol, Bristol, UK; c School of Physiology, Pharmacology & Neuroscience, University of Bristol, Bristol, UK; d Cancer Biology, Division of Cancer and Stem Cells, School of Medicine, University of Nottingham, Nottingham, UK

**Keywords:** Alternative splicing, vascular endothelial growth factor-A, splicing-sensitive fluorescent reporter, angiogenesis, mouse model

## Abstract

Vascular endothelial growth factor *(VEGF)-A* is differentially spliced to give two functionally different isoform families; pro-angiogenic, pro-permeability VEGF-A_xxx_ and anti-angiogenic, anti-permeability VEGF-A_xxx_b. *VEGF-A* splicing is dysregulated in several pathologies, including cancer, diabetes, and peripheral arterial disease. The bichromatic VEGF-A splicing-sensitive fluorescent reporter harboured in a transgenic mouse is a novel approach to investigate the splicing patterns of *VEGF-A in vivo*. We generated a transgenic mouse harbouring a splicing-sensitive fluorescent reporter designed to mimic *VEGF-A* terminal exon splicing (VEGF8ab) by insertion into the *ROSA26* genomic locus. dsRED expression denotes proximal splice site selection (VEGF-A_xxx_) and eGFP expression denotes distal splice site selection (VEGF-A_xxx_b). We investigated the tissue-specific expression patterns in the eye, skeletal muscle, cardiac muscle, kidney, and pancreas, and determined whether the splicing pattern could be manipulated in the same manner as endogenous *VEGF-A* by treatment with the SRPK1 inhibitor SPHINX 31. We confirmed expression of both dsRED and eGFP in the eye, skeletal muscle, cardiac muscle, kidney, and pancreas, with the highest expression of both fluorescent proteins observed in the exocrine pancreas. The ratio of dsRED and eGFP matched that of endogenous VEGF-A_xxx_ and VEGF-A_xxx_b. Treatment of the VEGF8ab mice with SPHINX 31 increased the mRNA and protein eGFP/dsRED ratio in the exocrine pancreas, mimicking endogenous *VEGF-A* splicing. The *VEGF-A* exon 8 splicing-sensitive fluorescent reporter mouse is a novel tool to assess splicing regulation in the individual cell-types and tissues, which provides a useful screening process for potentially therapeutic splicing regulatory compounds *in vivo*

## Introduction

The alternative splicing of pre-mRNA is the key driver of proteome diversity as it increases the coding capacity of a single gene [1]. Alternative splicing events are heavily regulated at different developmental stages, in different tissues and cell-types, under different conditions. A well-studied example of this regulated splicing is that of exon 8 of the vascular endothelial growth factor A *(VEGF-A)* gene. Within exon 8, use of a proximal 3’ splice site (PSS) gives rise to the canonical pro-angiogenic family of VEGF-A_xxx_ isoforms (VEGF-A_165_, VEGF-A_121_, VEGF-A_181_, etc., with the number denoting the number of amino acids). In 2002, Bates et al. [2] characterized a novel distal 3’ splice site (DSS) in exon 8, which resulted in a new family of anti-angiogenic isoforms, termed VEGF-A_xxx_b, the most dominant being VEGF-A_165_b. The VEGF-A_xxx_b family differ in the C-terminus by six amino acids, which results in the VEGF-A_xxx_b isoforms not being able to phosphorylate VEGF receptor 2 (VEGFR2) [3,4].

The role of *VEGF-A* exon 8 splicing in the pathogenesis of multiple disease-types has been studied, such as cancer, macular degeneration, nephropathy, preeclampsia, and ischaemic limb disease [2,5–8]. In many of these diseases, the pro-angiogenic VEGF-A_xxx_ isoform has been shown to be detrimental and therapeutic studies have focused on shifting the splicing ratio to increase VEGF-A_xxx_b/VEGF-A_xxx_ [5,6,9]. On the other hand, increased VEGF-A_165_b was reported to be detrimental in a mouse model of ischaemic limb disease [10].

The use of bichromatic splicing-sensitive fluorescent reporters is a novel method used to visualize splicing outcomes in living cells. It relies on the splice-site regulated expression of two fluorescent proteins from a single reporter. Such a method was first reported as a high-throughput cell-based screen of alternative splicing, which also enables quantitative single-cell analysis of alternative splicing [11]. This technique, although with monochromatic reporters, has also been used *in vivo* to assess *FGFR2* exon IIIc splicing as an indicator of mesenchymal epithelial transitions in prostate tumours [12]. Furthermore, Bonano et al. reported the insertion of the *FGFR2* exon IIIb silencing reporter into the *ROSA26* genomic locus of mice [13], and thus the ability to follow *FGFR2* alternative splicing in the whole organism. A bichromatic reporter designed on the backbone of the one used by Orengo et al. [11] to follow *FGFR2* exon IIIc splicing was also used to image the behaviour of cancer cells in xenografts and lung metastasis [14]. In addition, a bichromatic transgenic mouse model has also been generated to study the exclusion/inclusion of exon 22 in the Apt2a1 gene [15].

In the present study, we generated a VEGF-A exon 8 splicing-sensitive fluorescent reporter mouse (VEGF8ab) where dsRED expression denotes PSS selection (pro-angiogenic, pro-permeability VEGF-A_xxx_) and eGFP expression denotes DSS selection (anti-angiogenic, anti-permeability VEGF-A_xxx_b). We aimed to determine the reporter expression and splicing pattern in different tissues of the mouse and sought to validate whether the reporter expression mimicked endogenous *VEGF-A* splicing patterns by treating the mice with SPHINX 31, a potent and specific inhibitor of SRPK1 reported to increase VEGF-A_xxx_b relative to VEGF-A_xxx_ [16].

## Results

### Construction of the reporter mouse

**Figure 1.**
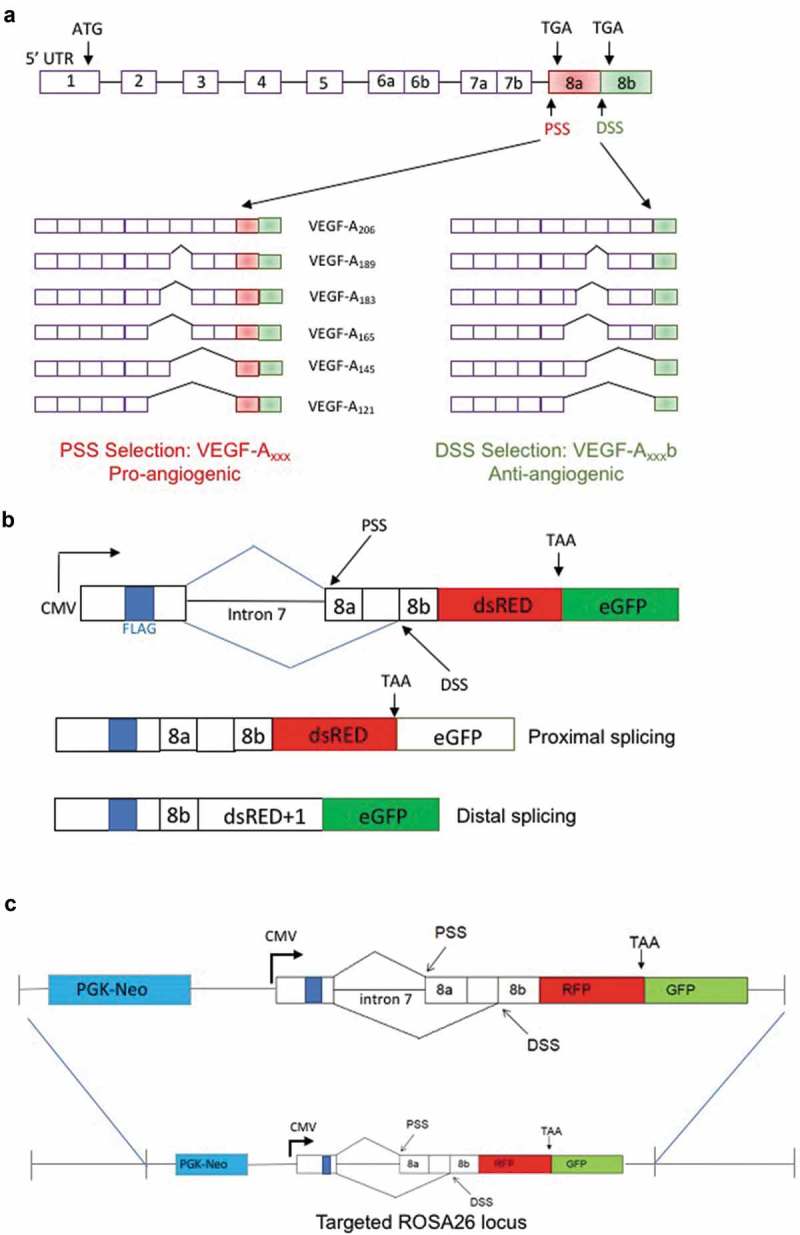
**VEGF-A exon 8 splicing reporter expression in transgenic mice**. (a) Alternative splicing of exon 8 of the endogenous VEGF-A pre-mRNA results in a pro-angiogenic and anti-angiogenic family of isoforms. (b) Schematic of the VEGF-A exon 8 splicing reporter construct with a CMV promoter. Use of the proximal splice site (PSS) results in the expression of dsRED; use of the distal splice site (DSS) puts dsRED in the +1-reading frame and eGFP in frame, resulting in the expression of eGFP fusion protein. A FLAG-tag is located before exon 7. (c) Targeting reporter constructs into the *ROSA26* genomic locus. Vectors harbouring the reporter were inserted into the *ROSA26* genomic locus by homologous recombination.

The VEGF-A exon 8 reporter was constructed in-house to mimic the alternative splicing events in exon 8 (Figure 1(a)) using the backbone of a previous bichromatic reporter designed for *FGFR2* splicing [14]. It contains a CMV promoter, followed by an artificial exon, which includes 11 bases of exon 7 of *VEGF-A* and a FLAG-tag. Next is intron 7 and exon 8 of the *VEGF-A* gene. The VEGF8ab reporter contains dsRED upstream of eGFP resulting in dsRED and eGFP being expressed from two different reading frames. Use of the exon 8 PSS results in the transcription of dsRED followed by a stop codon. Use of the exon 8 DSS, however, puts dsRED in the +1 reading frame; eGFP is now in-frame and is transcribed as a fusion protein (Figure 1(b)).

The VEGF-A exon 8 reporter mouse was generated by GenOway Ltd (France). Vectors harbouring the reporter were inserted into the *ROSA26* genomic locus by homologous recombination in embryonic stem cells (Figure 1(c)). Chimeras were screened for germline transmission by PCR and fluorescence imaging. DNA validation of insertion into the *ROSA26* locus is provided in Fig.S1. Homozygous mice (VEGF8ab^+/+^) on a mixed background were used for screening and experimental studies as they displayed better reporter expression.

### Imaging the expression of the VEGF-A exon 8 splicing reporter in multiple mouse tissues confirms it recapitulates the endogenous splicing patterns

PFA-fixed sections from the eye, heart, skeletal muscle, kidney, liver, spleen, lung, and pancreas, as well as retinal and choroid flat mounts of the eye, underwent microscopic analysis of reporter splice isoform expression. While there was very low levels of detection of either isoform in the liver, spleen, and lung, fluorescence was seen in the eye, kidney, pancreas and cardiac and skeletal muscle.

**Figure 2.**
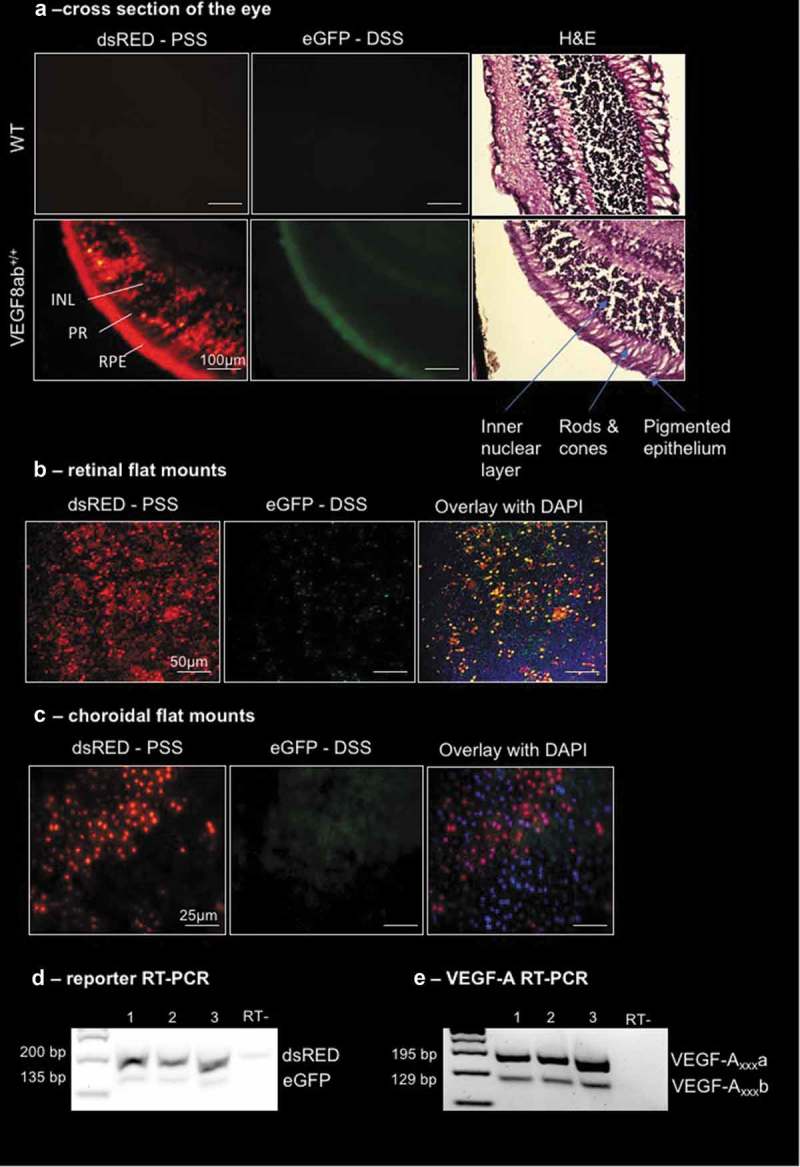
**VEGF-A exon 8 splicing reporter expression in the eye**. (a) Cross-section imaging of the eye from WT and VEGF8ab^+/+^ mice showed dsRED expression predominantly in the inner nuclear layer and the retinal pigmented epithelium (RPE). eGFP was found to be expressed only in the RPE (scale bar: 100 μm). haematoxylin and eosin (H&E) staining confirmed the localization of reporter expression. (b) Retinal flat mounts showed dsRED and eGFP expression in the retina (scale bar: 50 μm). (c) Choroid flat mounts showed only dsRED expression in the choroid (scale bar: 25 μm) (n = 10 mice). (d) RT-PCR for the VEGF-A reporter indicated higher expression of dsRED (201 bp) than eGFP (135 bp) in the eye, which mimicked the splicing pattern of the endogenous VEGF-A mRNA, where there was higher expression of VEGF-A_xxx_a (195 bp) than VEGF-A_xxx_b (129 bp), as shown in (e) (each lane equates to one mouse).

Cross-section imaging of the eye from VEGF8ab^+/+^ mice showed dsRED expression predominantly in the outer nuclear layer and the retinal pigmented epithelium (RPE). eGFP was found to be expressed predominantly in the RPE (Figure 2(a)), which correlates with endogenous patterns of expression as VEGF-A_165_b was previously reported to be expressed in RPE cells [17]. The localization of the expression of the reporter was determined by H&E staining and imaging of the same section. Retinal flat mounts showed both dsRED and eGFP to be expressed in the retina with some co-localization (Figure 2(b)). However, only dsRED was detected in the choroid of VEGF8ab^+/+^ mice (Figure 2(c)). This indicates that VEGF-A_165_b is predominantly expressed in the retina. Furthermore, when looking at the mRNA expression of both the reporter and endogenous VEGF-A in the eye, both dsRED and VEGF-A_xxx_a isoforms were expressed at a higher level than eGFP and VEGF-A_xxx_b (Figure 2(d,e)), indicating that the reporter mimics endogenous VEGF-A splicing.

**Figure 3.**
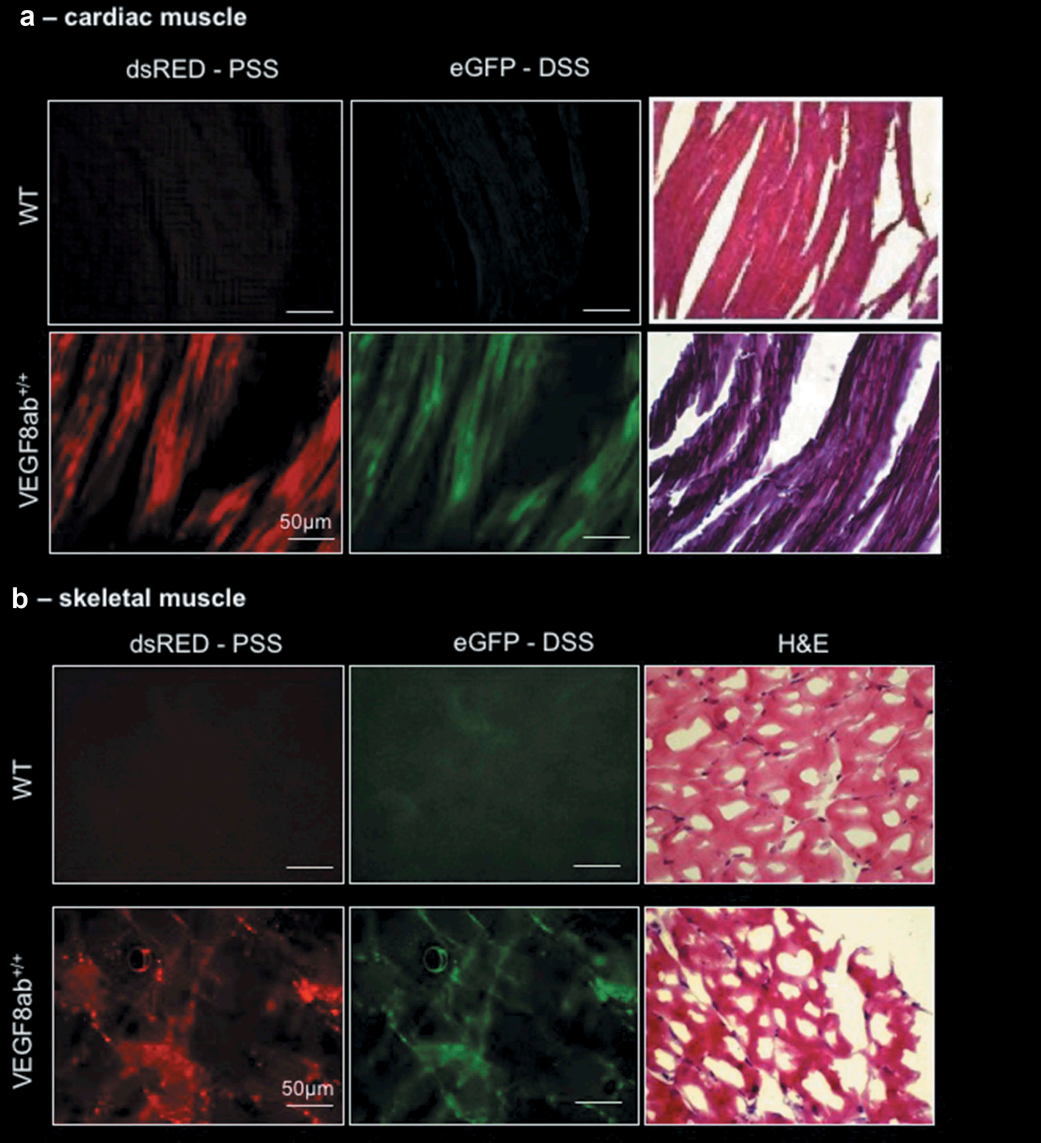
**VEGF-A exon 8 splicing reporter expression in cardiac and skeletal muscle**. (a) Fluorescence imaging of tissue sections from the left ventricle of WT and VEGF8ab^+/+^ mice indicated both dsRED and eGFP expression within the muscle fibres. The sections were also stained with haematoxylin and eosin (H&E) and to detect histological structures. (c) Fluorescence imaging of tissue sections from the skeletal muscle of WT and VEGF8ab^+/+^ mice indicated both dsRED and eGFP expression within the muscle fibres. H&E staining was then performed to detect histological structures (scale bar: 50 μm) (n = 10 mice).

To investigate expression in cardiac and skeletal muscle, tissue sections from the left ventricle and hind limb skeletal muscle of WT and VEGF8ab^+/+^ mice were imaged. Both dsRED and eGFP expression were detected within cardiac muscle fibres. (Figure 3(a)). Both dsRED and eGFP were also expressed in the skeletal muscle of VEGF8ab^+/+^ mice, with H&E staining confirming that both isoforms were expressed in both the muscle fibres and blood vessels (Figure 3(b)). These findings confirm that both splice isoforms of VEGF-A are generated within the cardiac and skeletal muscle [10].

**Figure 4.**
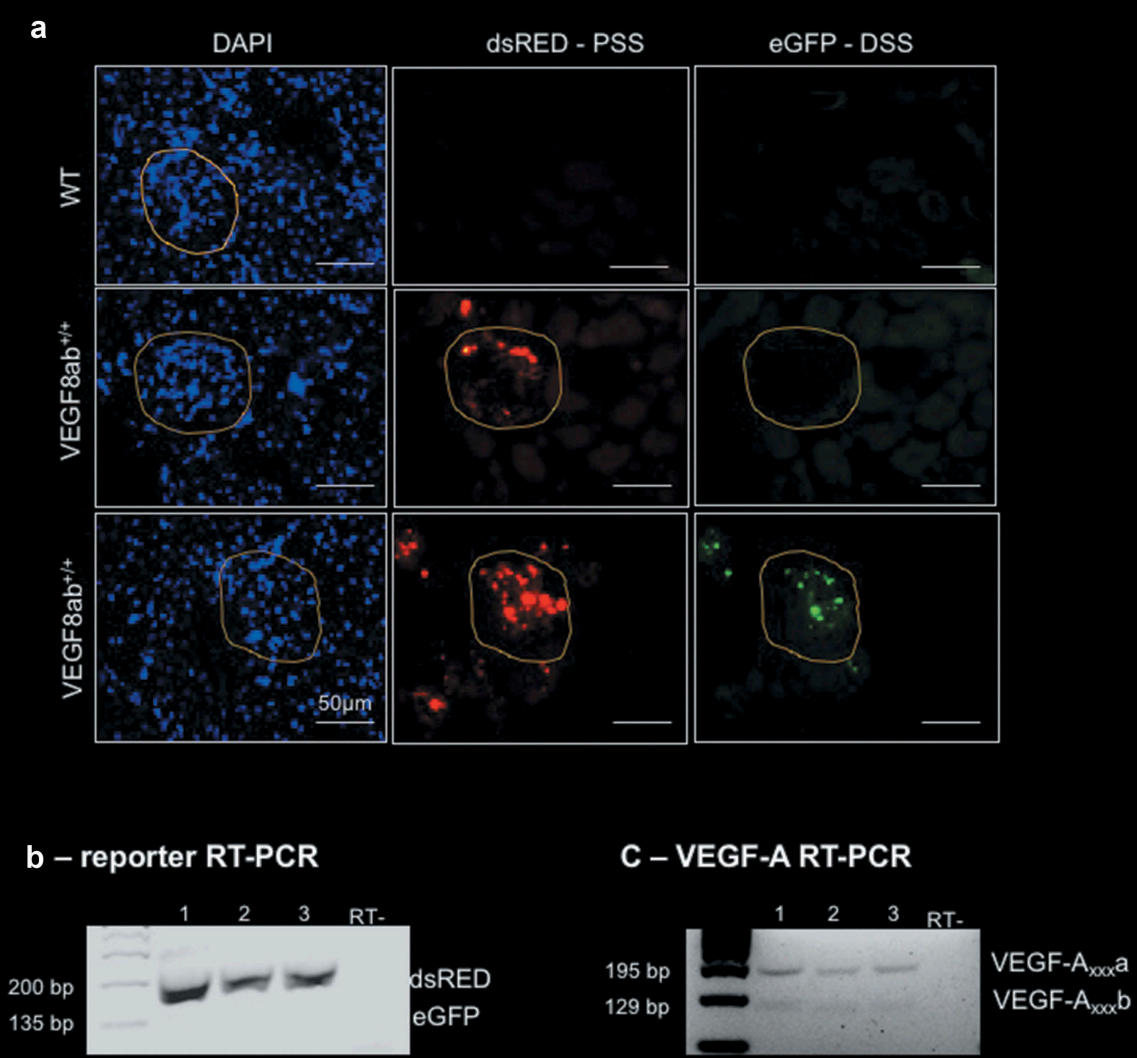
**VEGF-A exon 8 splicing reporter expression in the kidney**. (a) Fluorescence imaging of kidney sections from WT and VEGF8ab^+/+^ mice revealed reporter expression was localized to the glomeruli (circled in yellow). Some glomeruli only expressed dsRED (middle panel), whereas others expressed both dsRED and eGFP (lower panel; scale bar: 50 μm) (n = 10 mice). (b) RT-PCR for the VEGF-A reporter indicated higher expression of dsRED (201 bp) then eGFP (135 bp) in the renal cortex, which mimicked the splicing pattern of the endogenous VEGF-A mRNA, where there was higher expression of VEGF-A_xxx_a (195 bp) than VEGF-A_xxx_b (129 bp), as shown in (c) (each lane equates to one mouse).

VEGF-A_165_b was originally cloned from the human renal cortex, where it is predominantly expressed in the glomeruli [2]. When imaging the fluorescent reporter in the kidney, the reporter was found to be predominantly expressed in the glomeruli. Individual glomeruli varied in their expression with some expressing both dsRED and eGFP, whereas others only expressed dsRED (Figure 4(a); glomeruli circled in yellow), suggesting that individual glomeruli express different ratios of VEGF-A splice isoforms. This confirms previous observations we made that the variability of individual glomeruli permeability may be due to different ratios of the VEGF-A isoforms [6,18]. Furthermore, RT-PCR analysis of both the reporter and endogenous VEGF-A splice isoforms in the renal cortex revealed dsRED and VEGF-A_xxx_a to be most highly expressed, whereas no eGFP was detected and lower levels of VEGF-A_xxx_b (Figure 4(b,c)). This indicates that the reporter splicing pattern mimics that of endogenous VEGF-A in the renal cortex, although not exactly in this case as no eGFP could be detected with RT-PCR.

### Reporter expression and patterns in the pancreas

**Figure 5.**
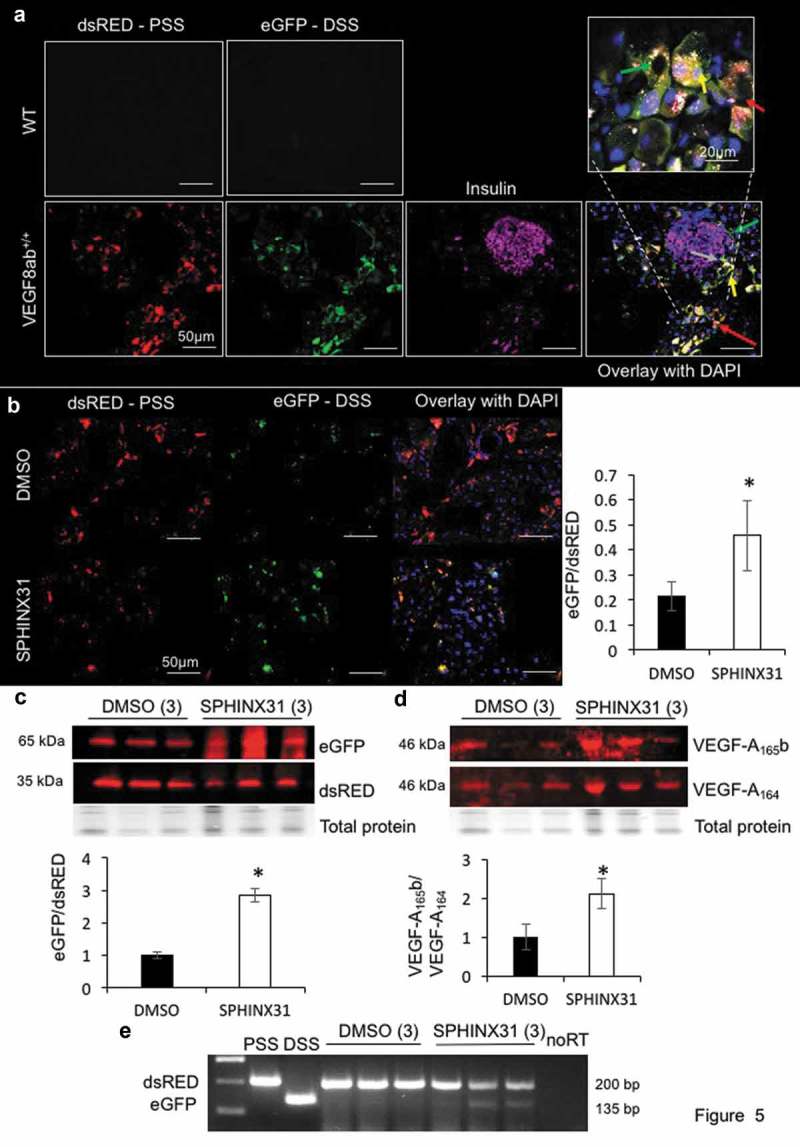
**VEGF-A exon 8 splicing reporter expression in the pancreas**. a. Fluorescence imaging of pancreatic section from WT and VEGF8ab^+/+^ mice showed high levels of dsRED and eGFP expression. The β cells within the pancreatic islets were stained with insulin, which showed that the VEGF8ab reporter was predominantly expressed in the acinar cells of the pancreas, and not the islets (scale bar: 50 μm) (n = 10 mice). Red arrow – predominantly PSS staining, green arrow predominantly DSS expression, yellow arrow = expression of both. Grey arrow, interstitial Cajal-like cell expressing GFP. Box, = high power image of pancreas. b. VEGF-A exon 8 splicing reporter mice were treated with either DMSO or SPHINX 31 (0.8 mg/kg) three times weekly for 3 weeks (intraperitoneal injection). SPHINX 31 significantly increased the eGFP/dsRED ratio in the pancreatic acinar cells, as quantified by fluorescence intensity (n = 3 mice, five fields of view from three areas of the pancreas in each mouse; *p < 0.05, Mann-Whitney test). c. Western blotting for the reporter FLAG-tag also showed a significant increase in the eGFP/dsRED ratio in the pancreas of SPHINX 31-treated mice, as normalized to the DMSO control mice (n = 3 mice; each lane equates to one mouse; *p < 0.05, Mann-Whitney test). d. Western blotting for endogenous VEGF-A splicing showed an increase in VEGF-A_165_b relative to VEGF-A_164_ in the pancreas of SPHINX 31-treated mice in comparison to DMSO controls (n = 3 mice; each lane equates to one mouse; *p < 0.05, Mann-Whitney test). e. RT-PCR indicated a shift in the splicing of the reporter to increase distal splice site (DSS) selection in SPHINX 31-treated mice in comparison to controls (note that these were different mice to those shown in C and D; each lane equates to one mouse).

In the pancreas, both dsRED and eGFP were detected (Figure 5(a)). Staining for insulin, a marker for pancreatic β cells, indicated that the reporter was expressed predominantly in the acinar cells of the exocrine pancreas; these cells can use the PSS (red arrow), DSS (green arrow), or both (yellow), and the epithelial cells of the ducts are able to express both isoforms.

### SRPK1 inhibition increased the eGFP/dsRED ratio as well as endogenous VEGF-A_165_b expression in the exocrine pancreas

To determine whether the splicing reporter responds to administration of small molecules splicing modulators in vivo, we administered SPHINX31 (an SRPK1 inhibitor shown previously to switch splicing towards increasing VEGF-A_165_b [16]) intraperitoneally in mice.

Fluorescent imaging of the exocrine pancreas of SPHINX 31-treated VEGF8ab^+/+^ mice showed a significant increase in the eGFP/dsRED ratio compared to DMSO-treated controls (Figure 5(b); p < 0.05). This was due to a change in splicing of the reporter: a decrease in dsRED and increase in eGFP. The reporter splicing was also assessed via Western blot analysis of the reporter Flag-tag expression in the pancreas. Both isoforms can be detected using this method as the dsRED equates to ~35 kDa and the eGFP fusion protein to ~65 kDa. The Flag-tag expression of each isoform also showed a significant increase in the eGFP/dsRED ratio in the pancreas of SPHINX 31-treated VEGF8ab^+/+^ mice compared to controls (Figure 5(c); p < 0.05). This switch in splicing was also confirmed at the mRNA level, with SPHINX 31-treated VEGF8ab^+/+^ mice showing an increased eGFP/dsRED ratio with RT-PCR (Figure 5(e)).

To determine whether the effect of SPHINX 31 on the VEGF8ab reporter splicing was mimicking the endogenous splicing of VEGF-A exon 8, we measured the VEGF-A_165_b/VEGF-A_164_ isoform expression ratio in the same protein from each mouse. The VEGF A20 antibody detects all VEGF-A isoforms, whereas the VEGF-A_165_b antibody is specific to the different c-terminus of VEGF-A_165_b. In agreement with the reporter splicing pattern, SPHINX 31 increased VEGF-A_165_b mRNA (Figure 5(d); p < 0.05) expression relative to VEGF-A_164_ when compared to DMSO-treated control mice.

## Discussion

We successfully generated a *VEGF-A* exon 8 splicing-sensitive fluorescent reporter mouse where dsRED expression denotes PSS selection (pro-angiogenic VEGF-A_xxx_) and eGFP expression denotes DSS selection (anti-angiogenic VEGF-A_xxx_b). We confirmed expression and splicing of the reporter in five tissues; eye, heart, skeletal muscle, kidney, and pancreas. The highest expression was observed in the pancreas and dsRED was the predominant protein expressed in each tissue assessed, as determined by the dsRED/eGFP ratio in each tissue, as well as by assessing the mRNA expression by RT-PCR. In addition, the splicing pattern of the reporter appeared to mimic that of the endogenous gene in the eye and kidney. Finally, we validated the efficacy of the VEGF8ab reporter *in vivo* with SPHINX 31, a potent SRPK1 inhibitor, which increased the eGFP/dsRED ratio in the pancreas. This change in the expression ratio of the two fluorescent proteins shows an increase in the use of the DSS and/or decrease in the use of the PSS, i.e. an increase in VEGF-A_xxx_b relative to VEGF-A_xxx_.

We chose to assess the VEGF8ab reporter expression in the eye, muscle, and kidney as these tissues have previously been widely reported to express the anti-angiogenic VEGF-A_165_b. In addition, we found that these tissues successfully expressed the reporter. Furthermore, we chose to assess the pancreas as this is where the reporter expression was the highest.

In the eye, we observed a high expression of dsRED (denoting the pro-angiogenic VEGF-A_xxx_) in the inner nuclear layer, photoreceptors, and the retinal pigmented epithelium, as well as in the vascular choroid. The expression of the pro-angiogenic VEGF-A is well-documented to be expressed by most ocular cell types, so this was not surprising. However, we only observed a detectable level of eGFP expression in the retinal pigmented epithelium. This result is in line with a previous study that confirmed the expression of VEGF-A_165_b in a retinal pigmented epithelial cell line [17], and in the developing retina [19]. The anti-angiogenic VEGF-A_165_b has been shown to be therapeutic in animal models of retinal pathologies, including diabetic retinopathy [20] and oxygen-induced retinopathy [21], as well as in experimental choroidal neovascularization [22]. The mRNA expression of both the reporter and endogenous VEGF-A in the eye revealed that both dsRED and VEGF-A_xxx_a were expressed at a higher level than eGFP and VEGF-A_xxx_b, indicating that the reporter mimics endogenous VEGF-A splicing.

The alternative splicing of exon 8 of *VEGF-A* has been reported in human and experimental peripheral arterial disease (PAD) where muscle ischaemia induces an angiogenic response that is frequently inadequate to meet the tissue perfusion needs [8]. This study showed that VEGF-A_165_b was elevated in muscle biopsies of human PAD, which inhibited angiogenesis and perfusion recovery in PAD muscle. Therefore, the observation of both dsRED and eGFP in the skeletal muscle of these mice is further evidence for the existence of *VEGF-A* exon 8 splicing in this tissue. The VEGF8ab reporter mouse would be a useful tool to assess *VEGF-A* exon 8 splicing in a mouse model of PAD, as described previously [10].

Although there are no reports of *VEGF-A* exon 8 splice isoform expression in the cardiac muscle, the circulating VEGF-A_xxx_/VEGF-A_xxx_b isoform balance has been well documented in both coronary artery disease (CAD) and acute myocardial infarction (AMI) [23,24]. In both instances, an increase in circulating VEGF-A_165_b levels was found to be associated with a worsened clinical outcome. The VEGF8ab reporter mouse indicates that the *VEGF-A* pre-mRNA may be alternatively spliced in the myocardium as both eGFP and dsRED expression was observed. Thus, this model has the potential to assess *VEGF-A* exon 8 splicing in the myocardium of experimental models of cardiac disease.

The role of VEGF-A_165_b in the kidney has been well-documented in recent years. Podocytes, glomerular visceral epithelial cells, have been reported to express high levels of both VEGF-A_165_ and VEGF-A_165_b [25]. This high expression of VEGF-A_165_b is suggested to be the reason for the lack of angiogenesis in the mature renal cortex as it has anti-angiogenic but cytoprotective properties [25]. Indeed, several mouse models of glomerular disease have been shown to have an increased expression of, or are exacerbated by the overexpression of, VEGF-A_164_; however, treatment with, or glomerular over-expression of, VEGF-A_165_b has been consistently shown to be reno-protective [6,18,26,27]. In the VEGF8ab reporter mouse, we also observed the expression of both eGFP and dsRED within the glomeruli, apparently expressed by the podocytes. Interestingly, we saw examples of glomeruli with mostly dsRED expression, as well as glomeruli that expressed both eGFP and dsRED from the same kidney. When assessing the glomerular permeability of individual glomeruli from the same kidney in previous reports, we observed a range of permeabilities that was suggested to be due to differing splicing patterns of *VEGF-A*, the dominant permeability factor in the kidney [6,18]. The splicing pattern of the reporter in the glomeruli of VEGF8ab mice in the present study provides further evidence for this suggestion, which should be explored further. RT-PCR analysis of both the reporter and endogenous VEGF-A splice isoforms in the renal cortex revealed dsRED and VEGF-A_xxx_a to be most highly expressed over eGFP and VEGF-A_xxx_b. This indicates that the reporter splicing mimics that of endogenous VEGF-A in the renal cortex, although we were not able to detect any eGFP mRNA in the renal cortex, which indicates that the reporter does not fully match the expression levels of the endogenous isoforms.

Within the pancreas of the VEGF8ab reporter mice, we saw very high expression of both dsRED and eGFP in the exocrine pancreas in comparison to the endocrine pancreas. This is evident in Figure 5 where co-staining with the pancreatic islet marker insulin revealed the reporter to be predominantly expressed in the acinar cells, and some cells showing much stronger GFP than dsRED staining. These cells look similar to the interstitial Cajal-like cells, previously described in the exocrine pancreas [28]. This was unexpected as much of the literature has focused on the role of VEGF-A in the development of pancreatic islets [29,30]. Furthermore, to the best of our knowledge, there have been no reports on the alternative splicing of VEGF-A in the pancreas. The reason why we see such high expression of the reporter in the exocrine pancreas is likely to be due to the use of the CMV promoter to drive the reporter, which has been previously reported to drive gene expression predominantly in the exocrine pancreas of mice [31]. This observation prompted us to use this tissue to assess the effects of SPHINX 31, a SRPK1 inhibitor known to increase the VEGF-A_xxx_b/VEGF-A_xxx_ splicing ratio [16]. Indeed, we found that SPHINX 31 increased the eGFP/dsRED ratio in the exocrine pancreas at both the mRNA and protein level, which reflected the increased endogenous VEGF-A_165_b/VEGF-A_164_ splicing ratio. Although the reporter was not a direct representation of the endogenous splicing pattern in every mouse, which may be due to the reporter having high expression in cell types that do not have a high expression level of endogenous VEGF-A, the same splicing switch was observed overall. In addition, validation via RT-PCR with primers specific for the VEGF-A splicing reporter isoforms confirmed that the switch in splicing was not due to programmed translational read-through to generate a protein known as VEGF-Axe [32]. Therefore, we can confirm that the VEGF8ab reporter mouse can be used to screen for compounds that affect *VEGF-A* exon 8 splicing *in vivo*. This novel tool allows for splicing regulation to be visualized in the individual cells types and tissues of a living organism.

While during the development of the reporter we ascertained that it spliced correctly by sequencing the RT-PCR products, we cannot completely exclude the possibility that a frameshift to a cryptic splice site or an error in translation may happen in a certain cell type or tissue and therefore give an artificial fluorescence read-out. However, in the tissues studied, we see a strong correlation of the behaviour of the reporter with the behaviour of the endogenous gene, which would be highly unlikely in the case of an artefact. Also, while being considered a strong promoter, it seems CMV does not drive expression of our reporter in some tissues. Variability of CMV expression in different cell types has been described before [33]. Ideally, several transgenic lines with different promoters could be developed, but this is at the moment cost-prohibitive.

In conclusion, we developed a *VEGF-A* exon 8 splicing-sensitive fluorescent reporter mouse (VEGF8ab) and examined the expression of dsRED and eGFP in the eye, skeletal muscle, cardiac muscle, kidney, and pancreas. We found that the reporter splicing pattern mimics that of endogenous VEGF-A exon 8 splicing when using SPHINX 31 to manipulate *VEGF-A* splicing in the pancreas. This model could be useful in the assessment of potentially therapeutic splicing regulatory compounds *in vivo*.

## Materials and methods

### Generation of the reporter construct

The VEGF-A splicing reporter was based on the pRG6 bichromatic splicing reporter, which contains restriction sites that allow for the insertion of the relevant sequences of the VEGF-A gene; a 2,699bp fragment containing the last 11 bases of exon 7, intron 7, and exon 8 of the VEGF-A gene. This DNA fragment was cloned into the RG6 plasmid backbone between XbaI and AgeI sites. The expression of the resulting PRG8ab reporter is under the control of a CMV promoter. Coding then begins with an artificial exon sequence. The stop codons of exons 8a and 8b were mutated to enable translation of the fluorescent proteins downstream. The reporter function relies on dsRED and EGFP being in mutually exclusive reading frames; proximal splice site selection results in dsRED being in-frame during translation, which is followed by a stop codon. On the other hand, distal splice site selection puts dsRED out of frame, resulting in dsRED+1 being translated, which forms a fusion protein with the now in-frame EGFP.

### Mice analysis

All experiments and procedures were approved by the UK Home office in accordance with the Animals (Scientific Procedures) Act 1986. Mice were maintained at the Biological Services Unit, University of Exeter, UK.

VEGF8ab^+/+^ mice and wild-type (WT) littermate controls were euthanized via cervical dislocation according to the Guide for the Care and Use of Laboratory Animals. Excised organs were washed in PBS and fixed overnight in 4% paraformaldehyde (PFA) at 4°C. Tissues were then embedded in OCT compound (Fisher Scientific) and frozen in dry ice before storing at −80°C. Frozen 10 μm-thick sections were cut onto poly-prep slides (Sigma Aldrich) using a cryostat, which were then air-dried for 30 min in the dark. Sections were fixed with 4% PFA for 10 min at room temperature, before rinsing three times in PBS for 5 min. Tissue sections were mounted with gel mount containing DAPI (VECTASHIELD). Images were taken using a Leica DM4000 B LED fluorescent microscope. In some instances, coverslips were removed from imaged slides and tissues underwent Haematoxylin and eosin (H&E) staining using standard methods.

### Treatment with SPHINX 31

Homozygous reporter mice were administered 0.8 mg/kg SPHINX 31 (kindly provided by Prof J Morris, UNSW) or DMSO vehicle via intraperitoneal injection three times weekly for 3 weeks (n = 3 per group). Mice were euthanized as described above. Three different parts of the pancreas were sectioned and imaged as described above. Pancreatic tissue was also collected for protein and mRNA analysis.

### Western blot analysis

Pancreatic tissue was homogenized in RIPA lysis buffer with protease inhibitors (both Fisher Scientific). Denatured protein samples were run on mini-PROTEAN® TGX Stain Free™ pre-cast gels (4–15%, BIORAD), which allow for visualization and accurate analysis of the total protein loaded for each sample using a Gel-Doc™ EZ (BIO-RAD) imaging system. The use of this system means a housekeeping protein loading control is not required as the amount of protein on the membrane for each sample can be quantified. Once protein had been transferred on to a PVDF membrane, total protein could be quantified. Membranes were blocked in 3% BSA in TBS plus 0.3% Tween before being probed with either anti-FLAG (Fisher Scientific), anti-mouse-VEGF-A_165_b (21^st^ Century Biochemicals), or anti-VEGF A20 (Santa Cruz), all at 1:1000 dilution in 3% BSA-TBS-Tween (0.3%), at 4°C overnight. As the anti-VEGF was raised in rabbit and the anti-mouse-VEGF-A_165_b was raised in mouse, we were able to probe for both proteins simultaneously. After washing membranes in TBS-Tween (0.3%), fluorescent secondary antibodies (LI-COR) were diluted in 3% BSA-TBS-Tween (0.3%), 1:10,000. Membranes were washed again and imaged with the LI-COR Odyssey® CLx. Analysis was performed using the Image Studio software (LI-COR), and the protein of interest was then normalized to the total protein loaded for each sample, as quantified by the Gel-Doc™ EZ imaging system (BIO-RAD).

### Immunofluorescence

Fixed pancreatic tissue sections were blocked with 3% bovine serum albumin (BSA) and 5% normal goat serum in PBS for 1 hr before incubating with the primary antibody (anti-insulin, 1:100, Cell Signalling) diluted in 3% BSA in PBS at 4°C overnight. After washing in PBS, the appropriate fluorescent secondary antibody was used (Alexa Fluor) in 3% BSA in PBS for 2 hr at room temperature. Sections were then washed in PBS before mounting with gel mount containing DAPI (VECTASHIELD). Images were taken using a Leica DM4000 B LED fluorescent microscope using a 40x objective, or a Leica DMi8 TCSP8 confocal using a 40x objective.

### RT-PCR analysis

Pancreatic tissue was immediately frozen on dry ice before RNA extraction was performed using the RNeasy Mini Kit (Qiagen). cDNA was then synthesized using the GoScript™ Reverse Transcription System (Promega). RT-PCR for the reporter was performed using primers positioned in the artificial exon and dsRED; F 5’-CATATGCCAAGTACGCCCCCTATTGACG-3’, R 5’-CTACAGGAACAGGTGGTGGC-3’. The PCR program consisted of 95ºC for 120 s, 35 cycles of 95ºC for 30 s, 55ºC for 30 s and 72ºC for 60 s, followed by 72ºC for 10 min. A band sized ~201 base pairs denotes PSS selection, and a band sized ~135 base pairs denotes DSS selection. Control plasmids that do not splice were used as positive controls for RT-PCR. RT-PCR for the VEGF-A splice variants was performed using primers positioned in exon 7 and THE 3’ UTR of exon 8b of the endogenous gene; F 5’-TTGTACAAGATCCGCAGACG-3’, R 5’-ATGGATCCGTATCAGTCTTTCCTGG-3’. The PCR program consisted of 95ºC for 120 s, 39 cycles of 95ºC for 60 s, 55ºC for 60 s and 72ºC for 60 s, followed by 72ºC for 10 min. This resulted in a PCR product for VEGF-A_xxx_b (129 bp) and VEGF-A_xxx_a (195 bp). All samples were run with negative controls (without reverse transcriptase; RT-).

### Statistical analysis

The statistical analysis was performed using GraphPad Prism software. Data was tested for normality and when found to be non-normally distributed, between-group comparisons were assessed with a Mann-Whitney test. All results are presented as the average ± standard error of the mean (SEM). Imaging and analysis was blinded to the researcher to restrict bias. P values <0.05 were considered statistically significant.
