# Supplementary material for: The VEGF-A exon 8 splicing-sensitive fluorescent reporter mouse is a novel tool to assess the effects of splicing regulatory compounds in vivo
Source: RNA Biol. 2019 Aug 21;16(12):1672–81. doi: 10.1080/15476286.2019.1652522 (PMC6844573; doi:10.1080/15476286.2019.1652522)
Supplement: Supplemental Material [file krnb-16-12-1652522-s001.zip › Supplementary information/Supplementary Figure 1.docx]

**Supplementary Figure 1. Representative Southern blot results to confirm homologous**

**recombination. A.** Schematic representation of the *ROSA26* wild-type and knock-in alleles.

**B.** The genomic DNA of the tested animals was compared to C57BL/6 wild-type genomic

DNA (WT).
